# Methylome Profiling in Fabry Disease in Clinical Practice: A Proof of Concept

**DOI:** 10.3390/ijms232012110

**Published:** 2022-10-11

**Authors:** Teodolinda Di Risi, Mariella Cuomo, Roberta Vinciguerra, Sara Ferraro, Rosa Della Monica, Davide Costabile, Michela Buonaiuto, Federica Trio, Ettore Capoluongo, Roberta Visconti, Eleonora Riccio, Antonio Pisani, Lorenzo Chiariotti

**Affiliations:** 1 CEINGE, Biotecnologie Avanzate, Via Gaetano Salvatore, 80145 Naples, Italy; 2 Department of Public Health, University Federico II, 80131 Naples, Italy; 3 Department of Molecular Medicine and Medical Biotechnologies, University Federico II, 80131 Naples, Italy; 4 SEMM-European School of Molecular Medicine, University Federico II, 80145 Naples, Italy; 5 Department of Clinical Pathology and Genomics, Azienda Ospedaliera per L’Emergenza Cannizzaro, 95126 Catania, Italy; 6 Institute for the Experimental Endocrinology and Oncology, Italian National Council of Research, 80131 Naples, Italy; 7 Institute for Biomedical Research and Innovation, Italian National Research Council, 90146 Palermo, Italy

**Keywords:** Fabry disease, methylome, Mendelian disease

## Abstract

Anderson–Fabry disease (FD) is an X-linked disease caused by a functional deficit of the α-galactosidase A enzyme. FD diagnosis relies on the clinical manifestations and research of *GLA* gene mutations. However, because of the lack of a clear genotype/phenotype correlation, FD diagnosis can be challenging. Recently, several studies have highlighted the importance of investigating DNA methylation patterns for confirming the correct diagnosis of different rare Mendelian diseases, but to date, no such studies have been reported for FD. Thus, in the present investigation, we analyzed for the first time the genome-wide methylation profile of a well-characterized cohort of patients with Fabry disease. We profiled the methylation status of about 850,000 CpG sites in 5 FD patients, all carrying the same mutation in the *GLA* gene (exon 6 c.901C>G) and presenting comparable low levels of α-Gal A activity. We found that, although the whole methylome profile did not discriminate the FD group from the unaffected one, several genes were significantly differentially methylated in Fabry patients. Thus, we provide here a proof of concept, to be tested in patients with different mutations and in a larger cohort, that the methylation state of specific genes can potentially identify Fabry patients and possibly predict organ involvement and disease evolution.

## 1. Introduction

Anderson–Fabry disease (FD) is a monogenic hereditary X-linked disease caused by mutations in the α-galactosidase gene (*GLA*), located on the long arm of the X chromosome (locus Xq22), resulting in the functional deficit of the α-galactosidase A (α-Gal A) enzyme [1]. More than 1000 *GLA* mutations have been identified so far, consisting of deletions, insertions, nonsense, missense (the majority), intron/exon junction, and splicing changing mutations [2]. α-Gal A activity defects lead to globotriaosylceramide (Gb3) storage in lysosomes mainly in vascular endothelium cells but also in various other cell types [3]. Thus, Fabry disease is a multisystem disease, and the most common symptoms are cutaneous (angiokeratoma), neurological (burning pain, hypo-, or hyperhidrosis), cerebrovascular (transient ischemic attacks, strokes), renal (proteinuria), and cardiovascular (cardiomyopathy, arrhythmia) [4]. Generally, symptoms develop early and severely in males, while in females, severity and progression are much more difficult to predict [5,6]. Regardless of sex, however, in all FD patients, age of disease onset, order of symptom onset, rate of disease progression, and comorbidities are highly heterogenous. *GLA* gene mutation analysis is a prerequisite for diagnosis, providing key information about the type of variant and less about its association with clinical manifestations. In fact, genotype/phenotype correlation is often unclear. The absence of a clear genotype/phenotype correlation is a consequence of several factors such as the high percentage of variants of uncertain (or unknown) significance (VUS) and the broad intra- and interfamily phenotypic heterogeneity associated with the same mutation. Thus, accurate diagnosis and clinical management are challenging, especially in the case of patients with no specific symptoms and with ambiguous genetic or biochemical profiles. Interestingly, a “red flag” approach has been proposed to increase the suspicion of rare diseases facilitating the diagnosis [7,8]. Recently, several studies have highlighted the importance of investigating genome-wide DNA methylation patterns for confirming the correct diagnosis of rare Mendelian diseases, taking advantage of the fact that affected individuals have unique and specific methylation signatures [9,10,11,12]. Moreover, methylation arrays, such as the Infinium Methylation EPIC Array (450k or 850k), have been useful also for classifying VUS as pathogenic or benign in rare Mendelian diseases [13,14]. Advantageously, diagnostic genome-wide DNA methylation analysis can be performed using peripheral blood because episignatures are early events during embryonic development and therefore present in numerous tissues, including blood [15,16,17,18,19]. In clinical practice, the extension of this approach to the management of suspected Fabry patients without typical symptoms and in the presence of VUS could be very helpful. To date, very few studies have addressed the role of DNA methylation in Fabry disease both at the gene-specific level and at the genome-wide level [20,21]. Thus, in the present study, we performed methylome analyses of DNA derived from peripheral blood cells of Fabry patients to investigate whether Fabry patients exhibit methylation profiles and signatures potentially useful for predicting disease progression.

## 2. Results and Discussion

### 2.1. Study Cohort

The study presented here included methylome analysis of peripheral blood from five male patients affected by Fabry disease (FD) and four nonaffected male relatives (CTRL). Patients selected for the present proof-of-concept study, aged between 26 and 56 years, from different families, carried the same mutation on exon 6 of the *GLA* gene c.901C>G (p.Arg301Gly), resulting in a nonconservative amino acid change in the encoded protein sequence. This variant has been classified as pathogenic (ClinVar, Global Variome shared LOVD, Varsome). We first confirmed that each of the five patients showed reduced α-Gal A enzymatic activity. All the selected patients presented renal impairment but variable cardiac, neurological and neuropathic symptoms. Patient genetic and clinical characteristics, including α-Gal A level of activity and type of therapy, are shown in Table 1.

### 2.2. Genome-Wide DNA Methylation Analyses in FD Patients and in Healthy Controls

**Figure 1:**
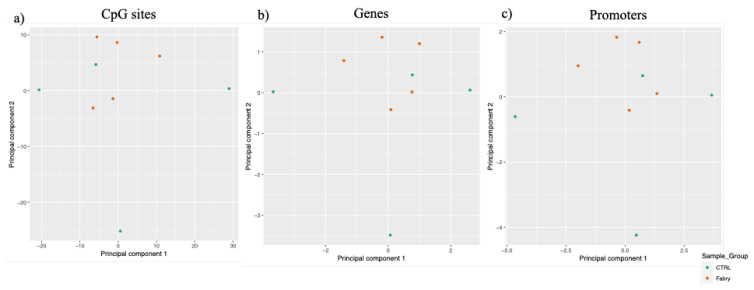
Principal Component Analysis for the Fabry Disease Group and CTRL Group. PCA plots showing the cluster of Fabry and CTRL groups in orange and in green, respectively. The plots are shown considering the DNA methylation levels at CpG sites (**a**), genes (**b**) and promoters (**c**).

The role of DNA methylation in the discrimination of Mendelian disorders is gradually emerging (reviewed in [10]). Thus, in the present study, we profiled genome-wide methylome using the Infinium EPIC array, including 850,000 probes for CpG methylation detection, in order to identify, for the first time, DNA methylation signatures associated with Fabry disease. Given the high amount of data derived from the genome-wide methylation analysis, we performed three different layers of bioinformatic analyses: as a first step, we compared patients affected by Fabry disease (FD) and the nonaffected control group (CTRL), considering all the quality filtered probes of the methylome. Then, we focused only on CpG sites, genes and promoters with the highest level of differences between groups. Finally, we went in depth by analyzing differentially methylated genes containing at least five differentially methylated CpGs. Hence, we analyzed IDAT files from the methylome array by using R-based RnBeads scripts [22,23]. After quality filtering, 692,016 probes were retained and subjected to subsequent analyses. Firstly, we evaluated whether FD patients were epigenetically different compared to healthy controls at the epigenome-wide level. To do this, we performed principal component analysis (PCA) by clustering samples according to the level of methylation at single CpG sites (Figure 1a) and at gene and promoter levels (Figure 1b,c, respectively). We observed that, in all analyzed regions and at all analyzed CpG sites, the comparative analyses of individual whole methylome profiles did not discriminate the FD group from the CTRL group. This may be partially explained by the small cohort of patients considered in the present preliminary study.

**Figure 2:**
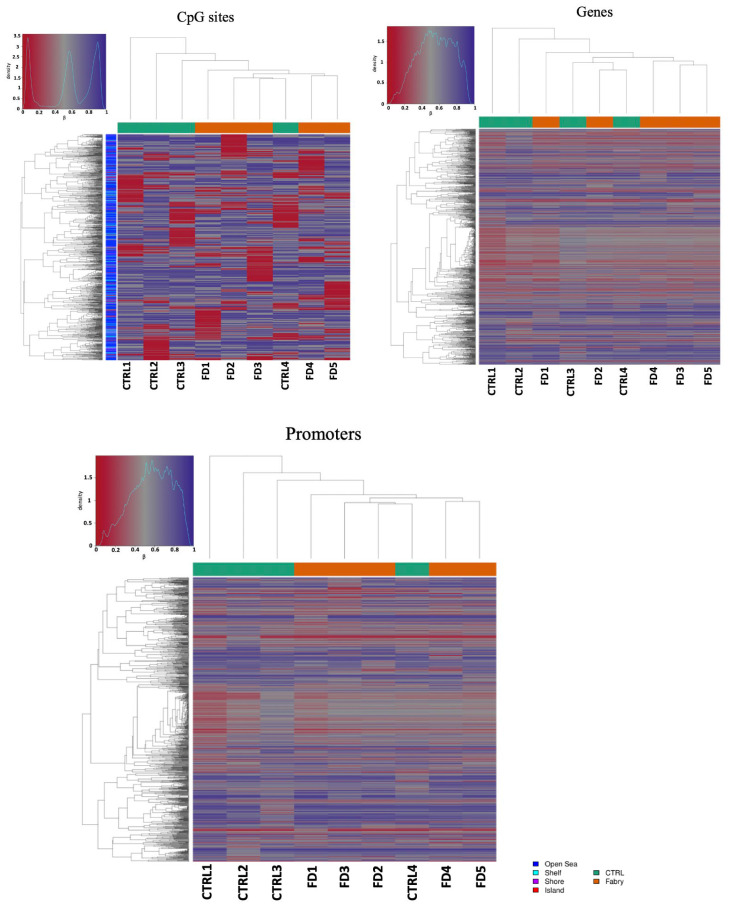
Hierarchical Cluster for the Fabry Disease group and CTRL Group. Heatmaps showing the methylation profiles at selected sites/regions with the highest variance across all samples.

We then performed hierarchical clustering based on the methylation levels at sites/regions with the highest variance across all samples. In doing so, we could select CpGs, promoters and genes that may be more discriminative between the FD group and the CTRL group. We found that especially at the CpG site and at promoter levels, a partial cluster of samples belonging to the FD group (in orange) and the CTRL group (in green) was present (Figure 2), with the exception of a nonaffected patient. These findings may indicate that, even though the global methylome was not sufficient to discriminate FD and CTRL groups, a deeper analysis allowed us to identify methylation signatures at given promoters and at specific CpG sites.

### 2.3. Identification of Differentially Methylated CpG Sites and Differentially Methylated Regions

**Figure 3:**
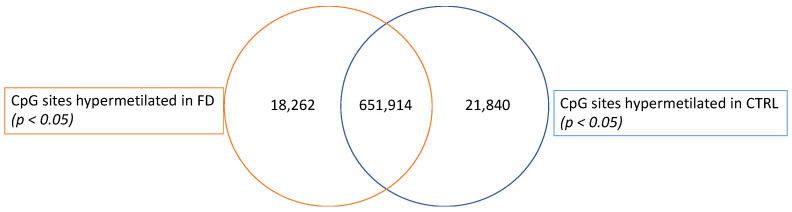
Venn diagram showing the statistically significant differentially methylated CpG sites in the FD and CTRL groups.

Since we did not observe strong differences in global DNA methylation levels between the FD and CTRL groups, we decided to investigate the methylation signals at CpG sites by considering the statistically significant differentially methylated CpG sites between FD and CTRL samples. Among the filtered 692,016 CpG sites, we found 18,262 CpG sites significantly (*p* ≤ 0.05) hypermethylated in the FD group and 21,840 CpG sites that were significantly (*p* ≤ 0.05) more methylated in the CTRL group (Figure 3).

Therefore, we focused on differentially methylated genes. We identified 189 differentially methylated genes (*p* ≤ 0.05) between the FD and CTRL groups. Among these genes, 99 genes were more methylated in FD compared to the CTRL group, while 91 genes were found less methylated in FDs with respect to CTRLs. We then filtered the hyper- and hypomethylated genes in the FD group that presented at least five CpG sites differentially methylated. The hyper- and hypomethylated genes in FD patients are listed in Table 2 and Table 3, respectively. Among the genes hypermethylated in FD patients, we found that 19 CpG sites were significantly hypermethylated in the ZFP57 gene. This gene is required for the maintenance of the germline-marked differential methylation at imprinting control regions [24] and has been associated with transient neonatal diabetes mellitus, a rare imprinting disorder [25].

## 3. Materials and Methods

### 3.1. Samples Processing

Peripheral human whole blood from 9 males (5 FD patients and 4 nonaffected controls) was collected and stored in tubes containing EDTA at 4 °C. Participants submitted a written consent form before blood was drawn. Human DNA was extracted from human whole blood with the QIAamp DNA Micro Kit (Qiagen, Hilden, Germany). Quality of DNA samples was assessed by NanoDrop 2000 (Thermo Scientific, Waltham, MA, USA). Bisulfite-converted DNA was used as input to the Illumina Infinium Human Methylation EPIC array 850k. Array data were generated according to the manufacturer’s protocol.

The study was approved by the Ethical Committee (Protocol Number: 181/19) of the University of Naples Federico II.

### 3.2. DNA Methylome Analyses

Methylome analyses were performed on genomic DNA extracted from the blood of each individual of the groups of patients described above. The methodologic approach consisted of Epic Array Illumina interrogating the methylation state of 850,000 CpG sites per sample. Bioinformatic analyses were performed on IDAT files by applying RnBeads R-based scripts [22,23]. As a first step, quality score was determined. According to sample annotations, batch effects and phenotype covariates were identified. DNA methylation distributions were analyzed, and intergroup and intragroup variability in methylation profiles were also identified. Furthermore, differential methylation between groups of samples was characterized. Differentially methylated CpG sites, promoters and CpG islands were calculated among single samples and among groups by Mann–Whitney tests, and heatmaps were generated. According to dissimilarities in terms of DNA methylation at each of the 850k CpG sites, a principal component analysis (PCA) was performed, and PCA plots were generated.

## 4. Conclusions

Although the low number of patients considered in the presented study does not allow us to identify specific episignatures able to discriminate patients and the clinical evolution of Fabry disease, we have provided for the first time a proof of concept that some genes are differentially methylated in patients, favoring the hypothesis that, as in other Mendelian diseases, methylome analysis may be of help for clinical management of FD. Moreover, some of these genes may also play a functional role in the different clinical manifestations of Fabry disease. Thus, it may be very interesting to deeply investigate how and if the different methylation statuses of these genes affect their expression levels.

## Figures and Tables

**Table 1:** Genetics and clinical characteristics of the study cohort. M = male; SNV = Single Nucleotide Variant.

Sample	Age	Sex	Gene Variant	Genomic Position (GRCh38)	Type and Classification of Variant	Interpretation of Variant (ClinVar-NCBI)	α-gal A Activity	Clinical Involvement	Therapy
FD1	56	M	c.901C>G p.Arg301Gly (EXON 6)	chrX: 101398468	SNV Missense	Pathogenic	Very Low	Renal, Cardiac and Neuropathic	Agalasidase alfa
FD2	26	M	c.901C>G p.Arg301Gly (EXON 6)	chrX: 101398468	SNV Missense	Pathogenic	Very Low	Renal and Cardiac	Agalasidase alfa
FD3	31	M	c.901C>G p.Arg301Gly (EXON 6)	chrX: 101398468	SNV Missense	Pathogenic	Undetected	Renal	Agalasidase alfa
FD4	43	M	c.901C>G p.Arg301Gly (EXON 6)	chrX: 101398468	SNV Missense	Pathogenic	Very Low	Renal, Cardiac and Neurological	Agalasidase alfa
FD5	38	M	c.901C>G p.Arg301Gly (EXON 6)	chrX: 101398468	SNV Missense	Pathogenic	Low	Renal, Cardiac and Neurological	Agalasidase alfa
CTRL1	38	M	-	-	-	-	-	-	-
CTRL2	42	M	-	-	-	-	-	-	-
CTRL3	32	M	-	-	-	-	-	-	-
CTRL4	48	M	-	-	-	-	-	-	-

**Table 2:** Differentially methylated genes with high degree of methylation in FD.

Gene Name	Average Methylation in FD (%)	Average Methylation in CTRL (%)	Number of Differentially Methylated CpGs within the Gene	Description
*ZFP57*	78.1	65.9	19	Zinc finger protein containing a KRAB domain. Diseases associated: diabetes mellitus, transient neonatal, 1 and transient neonatal diabetes mellitus.
*NUDT12*	50.7	43.8	7	Nudix hydrolase 12. Related pathways: NAD metabolism and nicotinate metabolism.
*AS3MT*	52	45.4	5	Arsenite methyltransferase. Catalyzes the transfer of a methyl group from S-adenosyl-L-methionine (AdoMet) to trivalent arsenical and may play a role in arsenic metabolism. Diseases associated: Borst–Jadassohn intraepidermal carcinoma and schizophrenia. Related pathways: metabolism and metapathway biotransformation phase I and II.
*RARRES2*	59.9	53.6	6	Retinoic acid receptor responder 2. Diseases associated: monckeberg arteriosclerosis and diabetes. Related pathways: Response to elevated platelet cytosolic Ca^2+^.
*PRSS41*	83.7	78.7	6	Serine protease 41. Predicted to enable sodium channel regulator activity. Predicted to be involved in proteolysis.
*ALOX15B*	60.3	63.2	10	Arachidonate 15-lipoxygenase type B. Encodes a member of the lipoxygenase family of structurally related nonheme iron dioxygenases involved in the production of fatty acid hydroperoxides. Diseases associated: autosomal recessive congenital ichthyosis and embryoma. Related pathways: arachidonic acid metabolism and prostaglandin and leukotriene metabolism in senescence.
*TOMM5*	58.9	56.4	5	Translocase of outer mitochondrial membrane 5. Located in mitochondrion, predicted to be involved in protein targeting to mitochondria. Diseases associated: Sengers syndrome. Related pathways: mitophagy and selective autophagy.
*CCNT1*	60.6	59.2	7	Cyclin T1. Encodes a member of the highly conserved cyclin C subfamily. The encoded protein associated with cyclin-dependent kinase 9 acts as a cofactor of human immunodeficiency virus type 1 (HIV-1) Tat protein and is necessary for full activation of viral transcription. Overexpression of this gene is implicated in tumor growth. Diseases associated: human immunodeficiency virus type 1 and human cytomegalovirus infection. Related pathways: male infertility and GPCR pathway.

**Table 3:** Differentially methylated genes with low degree of methylation in FD.

Gene Name	Average Methylation in FD (%)	Average Methylation in CTRL (%)	CpG Sites	Description
*FAM163B*	66	67.3	5	Family with sequence similarity 163 member B. Forecasted to be integral component of membrane.
*CREB3*	8.4	9.4	6	CAMP-responsive element-binding protein 3. Diseases associated: herpes simplex and ventricular tachycardia, catecholaminergic polymorphic, 2. related pathways: MIF mediated glucocorticoid regulation and development beta-adrenergic receptors regulation of ERK.
*FAM167B*	26	28	6	Family with sequence similarity 167 member B.
*PAPOLB*	57.5	59.5	6	Poly(A) polymerase beta. Allows polynucleotide adenylyltransferase activity, involved in mRNA polyadenylation. Forecasted to be located in endoplasmic reticulum and to be active in nucleus.
*LRRC24*	50.6	52.8	9	Leucine-rich repeat-containing 24. Predicted to act upstream of or within positive regulation of synapse assembly, to be integral component of membrane and to be active in extracellular matrix and extracellular space. Diseases associated: retroperitoneum carcinoma and atrial septal defect 2.
*BDH2*	60.1	62.4	5	3-Hydroxybutyrate dehydrogenase 2. Located in cytosol, allows 3-hydroxybutyrate dehydrogenase activity and NAD binding activity. Involved in epithelial cell differentiation and fatty acid beta-oxidation. Diseases associated: alpha-methylacetoacetic aciduria. Related pathways: ketone body metabolism and metabolism.
*ZNF429*	46.6	49	10	Zinc finger protein 429. It could allow DNA-binding transcription factor activity, RNA polymerase II-specific and RNA polymerase II cis-regulatory region sequence-specific DNA-binding activity. May be involved in regulation of transcription by RNA polymerase II and active in nucleus. Diseases associated: reflex sympathetic dystrophy. Related pathways: gene expression (transcription).
*KLK7*	54.3	58.3	13	Kallikrein related peptidase 7. The encoded protein may be involved in cancer invasion and metastasis, and increased expression of this gene is associated with unfavorable prognosis and progression of several types of cancer; has chymotrypsin-like activity and plays a role in the proteolysis of intercellular cohesive structures that precedes desquamation, the shedding of the outermost layer of the epidermis. Could be involved in the activation of precursors to inflammatory cytokines. Diseases associated: Netherton syndrome and dermatitis. Related pathways: extracellular matrix organization and collagen chain trimerization.
*ADRB1*	20.9	25	8	Adrenoceptor beta 1. The adrenergic receptors (subtypes alpha 1, alpha 2, beta 1 and beta 2) are a prototypic family of guanine nucleotide-binding regulatory protein-coupled receptors that mediate the physiological effects of the hormone epinephrine and the neurotransmitter norepinephrine. They are located primarily in the CNS, heart, coronary artery, kidney and muscle. Involved in the regulation of sleep/wake behaviors. Diseases associated: resting heart rate, variation in and short sleep, familial natural, 2. Related pathways: development ligand-independent activation of ESR1 and ESR2 and ADORA2B mediated anti-inflammatory cytokines production.
*PROB1*	22.2	26.8	10	Proline-rich basic protein 1. Located in nucleoplasm. Diseases associated: Hyperphenylalaninemia, Bh4-Deficient, A.
*FTH1P22*	81.6	87	5	Ferritin heavy chain 1 pseudogene 22.
*EGLN1P1*	35.6	41.6	5	Egl-9 family hypoxia-inducible factor 1 pseudogene 1.
*PLEKHA4*	54.1	60.4	12	Pleckstrin homology domain-containing A4. The encoded protein is a pleckstrin homology (PH) domain-containing protein. Elevated expression of this gene has been observed in some melanomas. Related pathways: PI metabolism and glycerophospholipid biosynthesis.
*TNNI3*	51	57.9	9	Troponin I3, cardiac type. This gene encodes the TnI-cardiac protein and is exclusively expressed in cardiac muscle tissues. Diseases associated: cardiomyopathy, dilated, 2A and cardiomyopathy, familial Hypertrophic, 7. Related pathways: beta-2 adrenergic-dependent CFTR expression and cytoskeletal Signaling.
*NKX1-1*	22.6	29.5	10	Encoded protein is a transcription factor of NKX family of homeodomain-containing proteins which are critical regulators of organ development. Diseases associated: orofaciodigital syndrome VIII and twin-to-twin transfusion syndrome.
*TNNT1*	31.3	40.4	7	Troponin T1, slow skeletal type. Encoded protein is a subunit of troponin, which is a regulatory complex located on the thin filament of the sarcomere. This complex regulates striated muscle contraction in response to fluctuations in intracellular calcium concentration. Diseases associated: nemaline myopathy 5 and nemaline myopathy. Related pathways: malignant pleural mesothelioma and striated muscle contraction pathway.

## Data Availability

The data presented in this study are available on request from the corresponding author.
